# SARS-CoV-2 Omicron variant spike protein maintains the ability to bind and activate human platelets: Comparison with the wild-type

**DOI:** 10.1038/s41598-026-46081-0

**Published:** 2026-04-09

**Authors:** Eriko Kusudo, Shuji Kawamoto, Tsuguhiro Matsumoto, Moritoki Egi

**Affiliations:** Department of Anesthesia, Kyoto University Hospital, 54 Shogoin Kawahara-cho, Sakyo-ku, Kyoto, Kyoto Japan

**Keywords:** COVID-19, Omicron, Platelet, Platelet activation, SARS-CoV-2, Spike protein, Diseases, Microbiology

## Abstract

Ongoing mutations in the SARS-CoV-2 spike protein contribute to the persistence of COVID-19. While the wild-type (WT) spike protein enhances platelet activity, the effects of the Omicron variant remain unclear. This study compared the effects of WT and Omicron spike proteins on platelet function in 10 healthy participants. We evaluated spike protein binding, P-selectin (platelet activation marker) expression, and aggregability using the Friedman test and Dunn’s post-hoc test. Results showed that both WT and Omicron spike proteins significantly increased binding to platelets and enhanced ADP-induced P-selectin expression compared to the control. However, neither protein significantly affected P-selectin expression under basal conditions or aggregation under low-dose (2 µM) ADP stimulation. Notably, under SFLLRN stimulation, only the Omicron variant significantly increased aggregation compared to the control, while the WT did not. No significant differences were observed between WT and Omicron across all assays. These findings suggest that the Omicron variant maintains a potent ability to modulate platelet reactivity, particularly through specific signaling pathways, despite numerous mutations. The qualitative nature of the spike protein-platelet interaction may play a crucial role in activation, highlighting the persistent prothrombotic risk across SARS-CoV-2 variants.

**Supplementary Information:**

The online version contains supplementary material available at 10.1038/s41598-026-46081-0.

## Introduction

COVID-19 is an infectious disease that has remained endemic worldwide for over six years. The infectivity of SARS-CoV-2 and its ability to evade immune responses are enhanced by mutations in the spike protein, a structural protein of the virus^1^. The spike protein binds to the host angiotensin-converting enzyme 2 (ACE2) receptor to facilitate infection^1^, making it a primary target for both prevention and treatment strategies against SARS-CoV-2.

Thromboembolism is a significant complication of COVID-19 and is associated with both disease severity and mortality^2^. Early studies during the pandemic reported a high incidence of thromboembolic events in patients with COVID-19, with rates ranging from 20% to 50%^2–5^. Furthermore, numerous reports have documented increased platelet activation and coagulability in these patients^6–11^. Zhang et al. demonstrated that the SARS-CoV-2 spike protein can activate platelets^12^, likely through binding to platelet ACE2. In their study, platelet aggregability was significantly elevated at spike protein concentrations of ≥ 0.5 µg/mL, and the platelet activation marker P-selectin was significantly elevated at 2 µg/mL^12^. However, direct binding of the spike protein to platelets was not demonstrated, and their study focused on the wild-type strain before the emergence of viral variants.

Currently, nearly all circulating SARS-CoV-2 variants are sub-strains of the Omicron lineage^13^. At the time of this study, no reports had addressed the effects of Omicron-derived spike proteins on platelet activity. We previously reported that spike proteins from the Alpha, Beta, Gamma, and Delta variants do not directly affect platelet activity^14^. Based on these findings, we hypothesized that the Omicron variant-derived spike protein would similarly have no direct effect on platelet activity. To test this hypothesis, we examined the binding and activation effects of the Omicron spike protein on platelets.

## Results

Blood samples were collected from 12 selected participants between October and November 2023. Two participants withdrew just prior to blood collection; thus, data were obtained from 10 participants. Voluntary responses regarding the timing of their final COVID-19 vaccination are shown in Supplementary Table S1. We measured (1) binding of the spike protein to platelets, (2) expression of P-selectin, a platelet activation marker, and (3) platelet aggregability, a key platelet function. (1) Binding and (2) P-selectin expression were expressed as mean fluorescence intensity (MFI) in flow cytometry, while (3) platelet aggregability was represented by the maximum aggregation rate in the light transmission assay. Platelet counts (×10^9^/L) in the control, W, O, and positive control groups were 174.0 (148.3–199.5), 172.5 (127.8–192.8), 174.0 (124.3–202.0), and 167.5 (137.3–191.5), respectively. Mean platelet volumes (MPV) (fL) were 7.30 (6.90–7.75), 7.60 (7.25–7.75), 7.50 (7.25–7.90), and 7.70 (7.30–7.80), respectively. Other raw data are shown in Supplementary Table S2.

### Spike protein binding to platelets

**Fig. 1:**
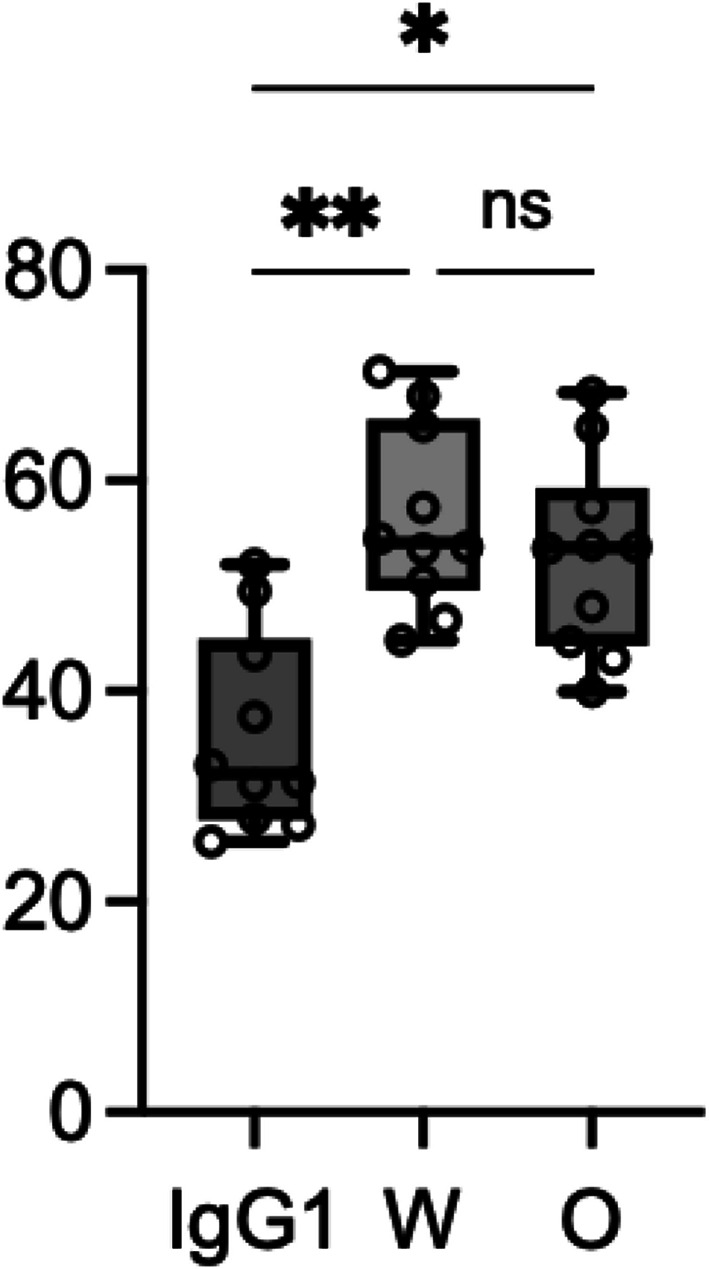
Spike protein binding to platelets. Quantitative comparison of spike protein binding (MFI) among the control (IgG1), wild-type (W) and Omicron (O) groups. In the box-and-whisker plot, the center line represents the median, the whiskers indicate the minimum and maximum values, and the open circles represent individual data points (*n* = 10). Both W and O groups showed significantly higher binding compared to the control, whereas no significant difference was observed between W and O. *IgG1:* isotype control, *W* wild-type, *O* Omicron variant, *MFI* mean fluorescence intensity, *ns* not significant; **p < 0.01, *p < 0.05 (Friedman test followed by Dunn’s post-hoc test).

The MFI was 32.17 (27.7–44.94) in the IgG1 group, 54.09 (49.55–65.87) in the W group, and 53.62 (44.29–59.33) in the O group (Fig. 1). Both groups W (*p* = 0.001) and O (*p* = 0.042) had significantly higher MFIs than IgG1. There was no significant difference between groups W and O. Representative flow cytometry histograms showing the rightward shift in MFI for groups W and O compared to IgG1 are presented in Supplementary Fig. S1.

### P-selectin expression

**Fig. 2:**
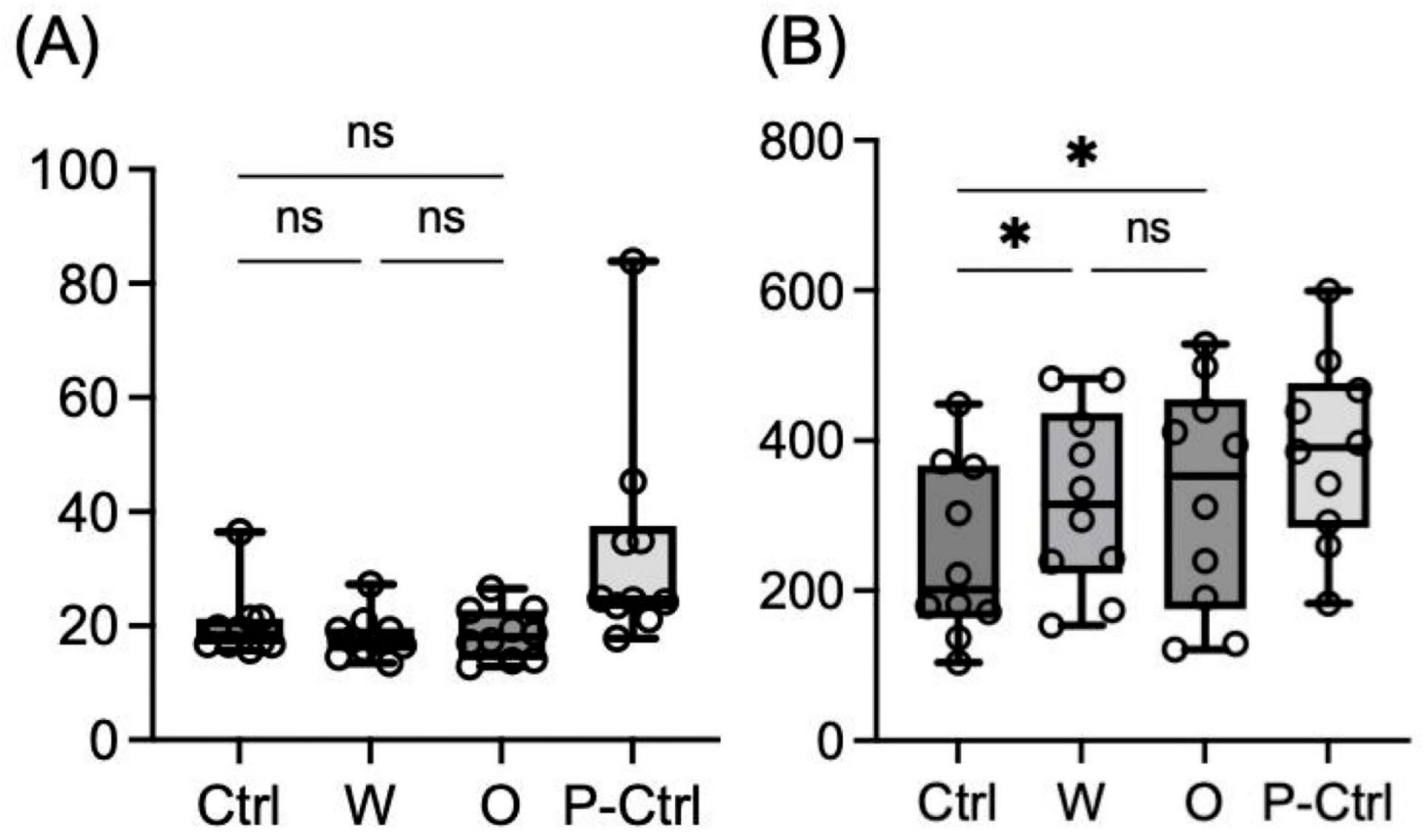
P-selectin expression on platelets. P-selectin expression measured as MFI under (**A**) basal conditions (no agonist) and (**B**) 20 µM ADP stimulation. In the box-and-whisker plot, the center line represents the median, the whiskers indicate the minimum and maximum values, and the open circles represent individual data points (*n* = 10). While no significant differences were observed under basal conditions, both W and O spike proteins significantly enhanced P-selectin expression following ADP stimulation compared to the control. There was no significant difference between W and O. *Ctrl* control (BSA), *W* wild-type, *O* Omicron variant, *P-Ctrl* positive control (20 µM adrenaline), *ADP* adenosine diphosphate, *MFI* mean fluorescence intensity, *ns* not significant; **p < 0.01; *p < 0.05 (Friedman test followed by Dunn’s post-hoc test).

P-selectin expression under basal conditions and ADP (20 µM) stimulation is shown in Fig. 2A and B, respectively. Under basal conditions, MFI was 18.28 (16.74–21.27) for the control, 17.36 (15.53–19.63) for group W, 18.05 (14.00–22.79) for group O, and 24.67 (22.93–37.51) for the positive control. No significant differences were observed among groups (excluding the positive control group). Under ADP stimulation, the P-selectin MFI was 201.4 (161.3–367.1) for the control, 315.5 (222.5–436.3) for group W, 352.8 (175.3–454.7) for group O, and 391.3 (283.6–476.6) for the positive control. Both groups W (*p* = 0.046) and O (*p* = 0.046) showed significantly higher MFIs compared to the control, but no significant difference was observed between groups W and O.

### Platelet aggregability

**Fig. 3:**
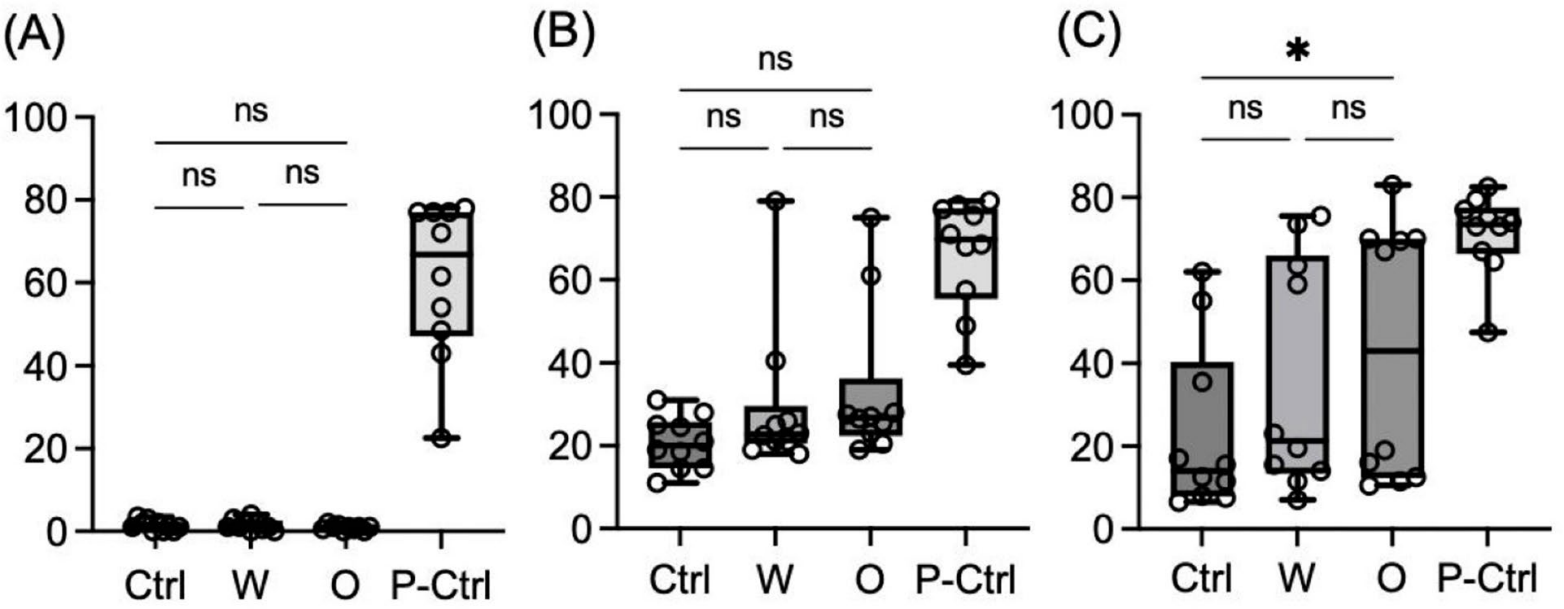
Platelet aggregability. Maximum platelet aggregation rates (%) evaluated (**A**) without stimulation, (**B**) under 2 µM ADP stimulation, and **(C)** under 1.4 µM SFLLRN stimulation. In the box-and-whisker plot, the center line represents the median, the whiskers indicate the minimum and maximum values, and the open circles represent individual data points (*n* = 10). (**C**) Under SFLLRN stimulation, only the Omicron variant (O) showed a significant increase in aggregation compared to the control, whereas the wild-type (W) did not. No significant difference was found between W and O. *Ctrl* control (BSA), *W* wild-type, *O* Omicron variant, *P-Ctrl* positive control (20 µM adrenaline), *ADP* adenosine diphosphate, *SFLLRN* protease-activated receptor (PAR)−1 agonist, *ns* not significant; **p < 0.01; *p < 0.05 (Friedman test followed by Dunn’s post-hoc test).

Maximum platelet aggregation rates under no stimulation, 2 µM ADP stimulation, and 1.4 µM SFLLRN stimulation are shown in Fig. 3A–C, respectively. Under no stimulation, platelet aggregation was almost zero in all groups except for the positive control. Under 2 µM ADP stimulation, the maximum aggregation rate was 20.00 (14.50–25.75) for the control, 22.75 (20.50–29.63) for group W, 26.75 (22.38–36.25) for group O, and 69.75 (55.38–77.25) for the positive control. No significant differences were observed among groups (excluding the positive control). Under 1.4 µM SFLLRN stimulation, the maximum aggregation rate was 14.00 (7.88–40.38) for the control, 21.25 (13.38–66.00) for group W, 43.00 (12.25–70.00) for group O, and 73.50 (66.38–77.63) for the positive control. Group O showed a significantly higher aggregation rate compared to the control (*p* = 0.017). However, no significant differences were observed between group W and the control or between groups W and O.

### Individual differences

Given the wide variety in P-selectin expression (MFI) and aggregation rates under agonist stimulation (Supplementary Table SS2), the ratio relative to the control was calculated for groups W and O (Supplementary Table SS3). Notably, participants who showed elevated aggregation rates did not overlap with those exhibiting increased P-selectin expression. In addition, participants who showed marked differences in aggregation under ADP stimulation were not the same as those under SFLLRN stimulation.

## Discussion

In this in vitro pilot study, we investigated the direct effects of SARS-CoV-2 wild-type and Omicron variant spike proteins on human platelet binding and function. Our findings demonstrate that both proteins significantly bind to platelets and modulate their activity, although the extent of activation appears to be dependent on the specific signaling pathway and agonist used. Notably, while both spike proteins significantly enhanced P-selectin expression under ADP stimulation, their impact on platelet aggregation was observed only under specific conditions—specifically with the Omicron variant under SFLLRN stimulation. Importantly, no statistically significant differences were observed between the wild-type and Omicron variant across any of the binding or activation assays. These results suggest that the Omicron variant, despite its numerous mutations, maintains a potent ability to modulate platelet reactivity comparable to that of the wild-type spike protein.

The mechanisms underlying SARS-CoV-2 spike protein-induced platelet activation have been increasingly elucidated. While early studies focused on the role of ACE2^12^, more recent evidence suggests that the spike protein can directly interact with alternative receptors on the platelet surface, such as CD42b (GPIbα)^15^ and TLR4^16^. These interactions trigger downstream intracellular signaling, including the activation of p38 MAPK and Akt pathways, which promote platelet degranulation and enhanced aggregation^12^.

The choice of analytical metrics for P-selectin expression warrants consideration. In our experimental setting, CD62P fluorescence showed a continuous distribution rather than a separable bimodal pattern (Supplementary Fig. S2). Therefore, we utilized MFI to accurately capture the population-wide “priming” or “sensitizing” effect of the spike protein, as this metric is highly sensitive for detecting shifts in the entire platelet population^17,18^.

B.1.1.529, the parental strain of Omicron, carries as many as 32 mutations in its spike protein^19^. In contrast, B.1.617.2 (Delta variant), which was prevalent worldwide until just before the emergence of Omicron, has only 10 mutations in its spike protein^20^, highlighting the remarkable number of mutations in Omicron. The receptor-binding domain (RBD) of the spike protein binds to ACE2 and plays an important role in SARS-CoV-2 entry into host cells^21^. Half of the mutations in the Omicron spike protein are located in the RBD, most near the ACE2-RBD binding interface^19^. However, this study found no difference in binding intensity to platelets or platelet activity between the Omicron and the wild-type.

At the time this study began, there were no reports on the effects of the Omicron spike protein on platelet activity, but several were published during our study period^22–24^. One study investigating spike protein binding to platelets showed that neither the wild-type nor Omicron spike proteins demonstrated significant binding compared with the control^22^. However, our study showed that both the wild-type and Omicron spike proteins exhibited significant binding, although no difference was observed between the two groups. This may provide important clues for future research on spike protein–platelet interactions. Vettori et al.^23^ reported that spike proteins derived from the Omicron variant increased platelet aggregation, and Sevilya et al.^24^ found that P-selectin expression was significantly elevated in BA.1 and BA.2 (Omicron sublineages) compared with controls. This study is highly novel in that it compares both wild-type and Omicron-derived spike protein using the same samples.

Marked individual differences in platelet activity under agonist stimulation may be attributable to genetic polymorphisms in P2Y12 and the protease-activated receptor (PAR)−1, which are platelet surface receptors. P2Y12 binds to ADP, and PAR-1 binds to SFLLRN, each leading to platelet activation^25,26^. Genetic polymorphisms associated with increased ADP-induced platelet aggregation have been identified in P2Y12^25^. The 14A/T polymorphism in PAR-1 is associated with the platelet response to SFLLRN^26^. In addition, there are individual differences in ACE2 expression^27^, along with multiple ACE2 polymorphisms and mutations^28^. Some ACE2 variants are predicted to increase host susceptibility to SARS-CoV-2^29^. Therefore, differences in platelet activity among individuals may not be due solely to spike protein mutations. Further studies are needed to clarify the changes in platelet activity caused by COVID-19 or SARS-CoV-2 spike proteins.

The Omicron variant has been associated with less thrombosis than the Delta variant or the wild-type strain^30^, and the severity and mortality associated with Omicron infection are lower compared with the Alpha and Delta variants^31,32^. However, these observations cannot rule out the influence of COVID-19 vaccination, and it remains possible that vaccination, rather than intrinsic differences between viral strains, contributed to the reduced severity and mortality. In fact, severe thrombosis and death from Omicron infection have been reported in unvaccinated individuals^33^. The development of arterial thrombosis in mechanically ventilated patients with Omicron infection has also been associated with increased risk of death^34^. COVID-19 vaccination also affects platelet receptor expression; in unvaccinated COVID-19 patients, P-selectin expression increases with disease severity, but in vaccinated COVID-19 patients, P-selectin expression is similar to that in non-COVID-19 individuals^35^.

As of February 2026, updated vaccination rates for COVID-19 have not been announced in most countries. However, vaccination rates are generally thought to have declined compared with the early stages of the pandemic. In the United States and the European Union/European Economic Area (EU/EEA), vaccination rates have dropped substantially. In the U.S., adult vaccination rates peaked in 2021, with approximately 80% having received at least one dose by the end of that year^36^. However, as of January 24, 2026, only 17.3% of adults had received the latest (2025–2026) COVID-19 vaccine^37^, suggesting declining public interest in vaccination. Similarly, in the EU/EEA, early vaccination rates were high: by the end of 2021, the median cumulative vaccination uptake of the primary course among adults aged 60 and older reached 89%^38^. Yet the latest COVID-19 vaccination rate for people aged 60 and older (from August 2024 to March 2025) dropped to 8.7% (range: < 0.1–52.8%)^39^. This trend underscores growing vaccine fatigue despite the ongoing risk posed by COVID-19 and its variants. These declining vaccination rates may make COVID-19 a renewed public health threat. Furthermore, if vaccination is related to the onset and severity of COVID-19-associated thrombosis, the presence or absence of vaccination and the timing of vaccination relative to infection could also contribute to individual differences in the effect of SARS-CoV-2 spike protein on platelet function.

This study has several limitations. First, while we have discussed potential pathways such as GPIbα and TLR4 based on recent literature, the precise molecular mechanisms remain to be fully elucidated in our specific experimental setting. Second, although our in vitro design using PRP allowed us to focus on the direct effects of spike proteins, it does not fully replicate the complex in vivo environment. Specifically, our model lacks the interaction with vascular endothelial cells (and the associated tissue factor) as well as other cellular components of whole blood, such as leukocytes and erythrocytes, which play critical roles in the coagulation cascade and thrombus formation under physiological flow conditions. Third, the spike protein concentration was fixed at 2 µg/mL based on previous work^12^, and its relevance to actual clinical infection level remains uncertain. Fourth, regarding our flow cytometry analysis, we prioritized MFI as our primary metric. Because P-selectin expression followed a continuous distribution, MFI ensured a more consistent and comprehensive evaluation of the activation state compared to fixed-gate percentages, which are highly sensitive to arbitrary threshold placement. Finally, the use of blood from healthy volunteers inherently limited both the sample size and the volume of blood that could be collected per donor. These constraints restricted the number of assays performed, prevented dose-response experiments, and necessitated the use of specific agonists as controls within the limited sample volume. Despite these limitations, our findings provide important preliminary insights into the differential effects of wild-type and Omicron spike proteins on platelet activation.

## Conclusion

Our study demonstrates that 2 µg/mL of Omicron-derived or wild-type spike protein added to the blood of healthy individuals directly binds to and activates platelets. No statistically significant difference, however, was observed between the two proteins. These findings suggest that the platelet-activating effect of the SARS-CoV-2 spike protein is maintained across variants. The qualitative nature of the spike protein-platelet interaction may play a crucial role in platelet activation, independent of mutations in the receptor-binding domain. Our pilot study results provide new insights into the mechanisms of variant-specific platelet modulation and underscore the persistent risk of platelet-mediated complications across different SARS-CoV-2 strains.

## Materials and methods

### Ethics and participants

This study was approved by the Ethics Committee of our institution (approval date: 13 April 2023, approval number: R0978-2) and conducted in accordance with the Declaration of Helsinki. Healthy adult volunteers provided written informed consent prior to participation. Participants were confirmed to have not taken any medications for at least 14 days prior to blood collection. All participants were also asked to complete a voluntary questionnaire regarding their most recent COVID-19 vaccination (Supplementary Table S1). Because this was a pilot study, the blood volume that could be collected from each participant was limited. A total of 14.6 mL of venous blood was collected from the median cubital vein using a 21-gauge needle. The blood was collected into tubes containing 3.2% trisodium citrate (prepared from tri-sodium citrate dihydrate; Nacalai Tesque) as an anticoagulant.

### Selection of spike protein

The Omicron variant (B.1.1.529) was first identified in November 2021 and designated as a VOC by WHO^40^. It rapidly replaced previously circulating strains^40,41^, and by February 2022 accounted for over 99% of SARS-CoV-2 sequences deposited in Global Initiative on Sharing Avian Influenza Data (GISAID)^42^. Numerous sublineages have since emerged with regional variation^13^. For this study, the spike protein of the parental B.1.1.529 lineage was selected to allow evaluation of the representative Omicron background.

### Preparation of platelet-rich plasma

Platelet-rich plasma (PRP) was prepared to examine spike protein binding, platelet aggregability, and P-selectin expression. PRP was obtained by centrifuging of whole blood at 160×*g* for 10 min at 22 °C. Platelet-poor plasma (PPP) was subsequently prepared by centrifuging the remaining sample at 1,600 × *g* for 15 min at 22 °C. Both PRP and PPP were maintained at room temperature (22 °C) and used for assays within 3 h of collection.

### Experimental groups

Wild-type spike protein (Recombinant SARS-CoV-2 Spike His Protein, CF; Novus Biologicals; 10549-CV-100) or Omicron spike protein (Recombinant SARS-CoV-2 B.1.1.529 Spike His-tag Protein, CF; Novus Biologicals; 11060-CV-100) was added at a final concentration of 2 µg/mL in groups W and O, respectively. The concentration of 2 µg/mL was selected based on previous work^12^. Samples were incubated with the spike proteins, BSA or adrenaline for 5 min at 37 °C. The negative control was 4 µg/mL bovine serum albumin (BSA) (Pierce™ Bovine Serum Albumin Standard Ampules; Thermo Scientific™; 23209) and the positive control was 20 µM adrenaline (Bosmin^®^ Injection 1 mg; Daiichi Sankyo Company, Limited). An IgG1 isotype control (PE Mouse IgG1 κ Isotype Control: BD Biosciences; 349043) was used to detect background signals in the binding assay.

### Spike protein binding to platelets

Binding of wild-type and Omicron spike proteins to platelets was evaluated using flow cytometry (FACS Calibur and Cell Quest™ Pro v5.2 software, both BD Biosciences). After incubation with spike proteins for 5 min, samples were fixed with 1× CellFIX (1% formaldehyde; BD Biosciences) for at least 1 h at room temperature. Subsequently, platelets were incubated with antibodies for 1 h at room temperature in the dark to detect spike protein binding. Platelets were identified using peridinin chlorophyll protein (PerCP)-conjugated anti-CD61 antibody (1 µL/50 µL sample; BD Biosciences; 345056) and spike protein binding was detected with phycoerythrin (PE)-conjugated anti-SARS-CoV-2 spike S1 protein antibody (2 µL/50 µL sample; Novus Biologicals, LLC; FAB105403P). Results were expressed as the MFI. A total of 10,000 platelet events were gated by forward scatter (FSC), side scatter (SSC), and PerCP-labeled anti-CD61 antibody expression. The detailed gating strategy is illustrated in Supplementary Fig. S2.

### P-Selectin expression

P-selectin (CD62P) was measured using a PE-conjugated anti-CD62P (P-selectin) antibody (2 µL/50 µL sample; BD Biosciences; 348107) with the anti-CD61 antibody for platelet gating. The specific procedure was as follows: (1) 43 µL of PRP was distributed into eight groups. (2) 5 µL of PBS was added to four groups (no stimulation), and 5 µL of 200 µM ADP was added to the remaining four groups (final concentration 20 µM). (3) Each group then received 4 µg/mL of BSA, 20 µM of adrenaline, or 2 µg/mL of either wild-type or Omicron variant spike protein. (4) These mixtures were incubated for 5 min at 37 °C. Following this incubation, samples were fixed with CellFIX for at least 1 h at room temperature. Subsequently, the fixed platelets were incubated with the antibodies for 60 min at room temperature in the dark. Results were expressed as the MFI.

### Platelet aggregability

Platelet aggregability was assessed using PRP with PPP as the 100% reference for light transmission^43^. ADP (2 µM) and PAR-1 agonist SFLLRN (1.4 µM) were used as platelet agonists. Samples were pre-incubated with spike proteins, BSA, or adrenaline for 5 min at 37 °C prior to agonist stimulation. Aggregation was measured with the MCM HEMA TRACER 212 analyzer (MCM Medical) for 7 min, and the maximum aggregation rate was recorded. The preparation and subsequent measurements were performed at room temperature (22 °C). Results were expressed as maximal aggregation (%). Representative aggregation curves are shown in Supplementary Fig. S3.

### Statistical analysis

Results were presented as median with IQR. IgG1 served as the control for binding assays, and BSA served as the control for aggregability and P-selectin assays. To account for multiple comparisons among the three groups (Control, W, and O), the Friedman test was performed. When a significant difference was observed, Dunn’s post-hoc test was used for pairwise comparisons. A p-value < 0.05 was considered statistically significant. Statistical analyses were performed using GraphPad Prism software (ver. 10.6.1).

## Supplementary Information

Below is the link to the electronic supplementary material.

Supplementary Material 1

Supplementary Material 1

## Data Availability

All data generated or analyzed during this study are included in the published article and the accompanying electronic supplementary materials.
