# Supplementary material for: SARS-CoV-2 Omicron variant spike protein maintains the ability to bind and activate human platelets: Comparison with the wild-type
Source: Sci Rep. 2026 Apr 9;16:16753. doi: 10.1038/s41598-026-46081-0 (PMC13223238; doi:10.1038/s41598-026-46081-0)
Supplement: Supplementary file 1 — Supplementary Material 1 [file 41598_2026_46081_MOESM1_ESM.docx]

**Table S1** Vaccination timing calculated from the date of blood collection and number of vaccinations.

| Participant No. | Vaccination timing from the date of blood collection | Number of vaccinations |
| --- | --- | --- |
| 2 | 19 months ago | N/A |
| 3 | 9 months ago | N/A |
| 4 | 21 months ago | 3rd |
| 5 | 14 months ago | 4th |
| 6 | 18 months ago | N/A |
| 7 | 18 months ago | 3rd |
| 8 | 10 months ago | 5th |
| 9 | 21 months ago | 3rd |
| 10 | 14 months ago | 4th |
| 12 | 21 months ago | 3rd |

Several participants responded that the number of vaccinations was unknown.

**Table S2** Raw data for each assay.

| 1. Spike protein binding to platelets | | | | |
| --- | --- | --- | --- | --- |
| Participant No. | IgG1 | W | O |  |
| 2 | 27.89 | 46.77 | 44.7 |  |
| 3 | 27.31 | 50.48 | 43.06 |  |
| 4 | 31.39 | 44.8 | 39.94 |  |
| 5 | 25.72 | 68.01 | 57.44 |  |
| 6 | 32.94 | 53.68 | 53.69 |  |
| 7 | 43.42 | 53.48 | 53.54 |  |
| 8 | 31.11 | 57.42 | 53.8 |  |
| 9 | 37.58 | 70.34 | 65 |  |
| 10 | 52 | 65.15 | 68.34 |  |
| 12 | 49.48 | 54.5 | 48.02 |  |
| 2-1. P-selectin expression under basal conditions | | | | |
| Participant No. | Ctrl | W | O | P-Ctrl |
| 2 | 16.8 | 19.19 | 17.07 | 24.2 |
| 3 | 15.69 | 17.22 | 12.89 | 24.58 |
| 4 | 21.25 | 17.5 | 22.73 | 45.25 |
| 5 | 19.42 | 15.87 | 22.95 | 34.69 |
| 6 | 19.59 | 14.51 | 17.41 | 23.52 |
| 7 | 36.48 | 27.26 | 26.57 | 83.85 |
| 8 | 16.75 | 19.25 | 18.68 | 34.93 |
| 9 | 17.13 | 13.49 | 13.86 | 21.16 |
| 10 | 21.34 | 20.78 | 19.44 | 17.8 |
| 12 | 16.72 | 16.51 | 14.04 | 24.75 |
| 2-2. P-selectin expression under ADP 20 µM stimulation | | | | |
| Participant No. | Ctrl | W | O | P-Ctrl |
| 2 | 303.23 | 421.57 | 311.55 | 599.03 |
| 3 | 136.92 | 153.39 | 190.63 | 342.69 |
| 4 | 179.55 | 336.36 | 440.23 | 438.65 |
| 5 | 448.3 | 482.52 | 411.51 | 385.54 |
| 6 | 181.56 | 238.71 | 121.57 | 505.55 |
| 7 | 365.46 | 480.55 | 498.28 | 291.52 |
| 8 | 171.81 | 294.55 | 239.56 | 182.84 |
| 9 | 104.37 | 174.04 | 129.36 | 259.84 |
| 10 | 221.26 | 242.25 | 394.1 | 396.99 |
| 12 | 372.03 | 381.25 | 528.19 | 466.97 |
| 3-1. Aggregation rates without stimulation | | | | |
| Participant No. | Ctrl | W | O | P-Ctrl |
| 2 | 3.5 | 2.5 | 1.5 | 61.5 |
| 3 | 1 | 1 | 0 | 78 |
| 4 | 2 | 2 | 1 | 77 |
| 5 | 3 | 1 | 1 | 43 |
| 6 | 0 | 0 | 0.5 | 22.5 |
| 7 | 0 | 3 | 1 | 77 |
| 8 | 1 | 0 | 0.5 | 48.5 |
| 9 | 0 | 0.5 | 0 | 54 |
| 10 | 1 | 4 | 1 | 77 |
| 12 | 2 | 1 | 2 | 72 |
| 3-2. Aggregation rates under ADP 2 µM stimulation | | | | |
| Participant No. | Ctrl | W | O | P-Ctrl |
| 2 | 31 | 40.5 | 61 | 68.5 |
| 3 | 19 | 25 | 26.5 | 75.5 |
| 4 | 21 | 22.5 | 27.5 | 77 |
| 5 | 14.5 | 18 | 23 | 39.5 |
| 6 | 14.5 | 23 | 25.5 | 71 |
| 7 | 28 | 19 | 19 | 49 |
| 8 | 18.5 | 26 | 27 | 57.5 |
| 9 | 11 | 21 | 20.5 | 68 |
| 10 | 24.5 | 21 | 28 | 78 |
| 12 | 25 | 79 | 75 | 79 |
| 3-3. Aggregation rates under SFLLRN 1.4 µM stimulation | | | | |
| Participant No. | Ctrl | W | O | P-Ctrl |
| 2 | 55 | 59 | 70 | 73 |
| 3 | 11.5 | 73.5 | 69.5 | 79.5 |
| 4 | 62 | 75.5 | 83 | 75 |
| 5 | 15.5 | 15.5 | 16 | 47.5 |
| 6 | 7.5 | 19.5 | 19 | 82.5 |
| 7 | 17 | 23 | 70 | 73 |
| 8 | 35.5 | 63.5 | 67 | 64.5 |
| 9 | 6.5 | 11.5 | 11.5 | 67 |
| 10 | 8 | 7 | 10.5 | 74 |
| 12 | 12.5 | 14 | 12.5 | 77 |

This table includes the raw values for spike protein binding (MFI), P-selectin expression (MFI), and platelet aggregation rates (%). IgG1: isotype control; Ctrl: control (BSA); W: wild-type; O: omicron variant; P-Ctrl: positive control (20 µM adrenaline); ADP: adenosine diphosphate; SFLLRN: protease-activated receptor (PAR)-1 agonist; MFI: mean fluorescence intensity.

**Table S3** Ratio of P-selectin expression (MFI) or aggregation rate under agonist stimulation to the control.

| P-selectin expression under ADP 20 µM stimulation | | | Aggregation rates under ADP 2 µM stimulation | | | Aggregation rates under SFLLRN 1.4 µM stimulation | | |
| --- | --- | --- | --- | --- | --- | --- | --- | --- |
| Participant | W/Ctrl | O/Ctrl | Participant | W/Ctrl | O/Ctrl | Participant | W/Ctrl | O/Ctrl |
| 2 | 1.39 | 1.03 | 2 | 1.31 | 1.97 † | 2 | 1.97 † | 1.27 |
| 3 | 1.12 | 1.39 | 3 | 1.32 | 1.39 | 3 | 6.39 ‡ | 6.04 ‡ |
| 4 | 1.87 † | 2.45 ‡ | 4 | 1.07 | 1.31 | 4 | 1.22 | 1.34 |
| 5 | 1.08 | 0.92 | 5 | 1.24 | 1.59 † | 5 | 1 | 1.03 |
| 6 | 1.31 | 0.67 | 6 | 1.59 † | 1.76 † | 6 | 2.6 ‡ | 2.53 ‡ |
| 7 | 1.31 | 1.36 | 7 | 0.68 | 0.68 | 7 | 1.35 | 4.12 ‡ |
| 8 | 1.71 † | 1.39 | 8 | 1.41 | 1.46 | 8 | 1.79 † | 1.89 † |
| 9 | 1.67 † | 1.24 | 9 | 1.91 † | 1.86 † | 9 | 1.77 † | 1.77 † |
| 10 | 1.09 | 1.78 † | 10 | 0.86 | 1.14 | 10 | 0.88 | 1.31 |
| 12 | 1.02 | 1.42 | 12 | 3.16 ‡ | 3 ‡ | 12 | 1.12 | 1 |

†: more than 1.5; ‡: more than 2; ADP: adenosine diphosphate; W: wild-type; O: omicron variant; Ctrl: control (BSA); SFLLRN: protease-activated receptor (PAR)-1 agonist.

**Figure S1** Representative histograms of spike protein binding to platelets.


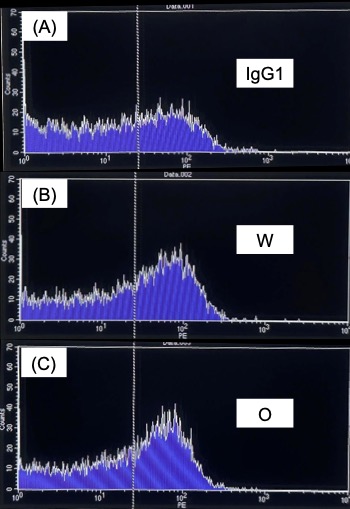


Representative flow cytometry histograms showing the binding of SARS-CoV-2 spike proteins to platelets from Participant No. 4. Platelet-rich plasma (PRP) was incubated for 5 minutes with 2 µg/mL of either wild-type or Omicron-derived spike protein. (A) IgG1: Isotype control showing background signals (MFI: 31.39). (B) W: Wild-type spike protein binding (MFI: 44.80). (C) O: Omicron variant spike protein binding (MFI: 39.94). The x-axis represents the fluorescence intensity of the PE-conjugated anti-spike antibody, and the y-axis indicates the cell count. A total of 10,000 platelet events were gated based on forward scatter, side scatter, and PerCP-labeled anti-CD61 antibody expression. Both wild-type and Omicron spike proteins demonstrated a rightward shift in fluorescence compared to the IgG1 control, indicating successful binding to the platelet surface. PRP: platelet-rich plasma; W: wild-type; O: Omicron variant; MFI: mean fluorescence intensity; PE: phycoerythrin; PerCP: peridinin chlorophyll protein.

**Figure S2** Gating strategy and representative histograms for P-selectin expression.


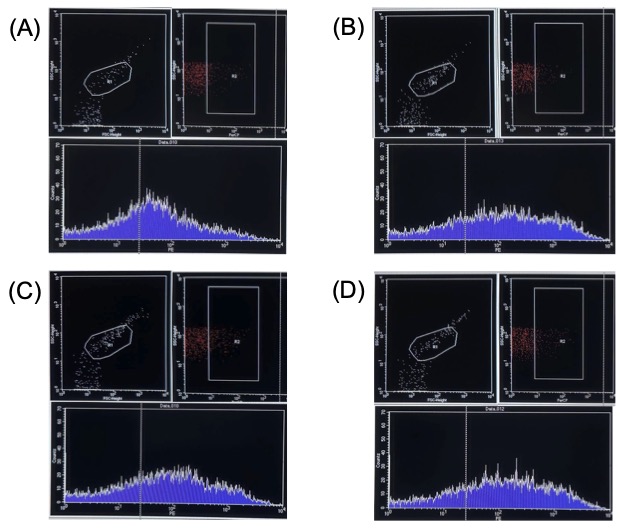


Flow cytometry gating strategy and representative histograms for P-selectin expression. Platelets were first gated based on forward scatter (FSC) and side scatter (SSC) characteristics (R1), and then identified by CD61-PerCP expression (R2). **(A, B)** Participant No. 4 under ADP stimulation: (A) Control (MFI: 179.55) and (B) Omicron variant (MFI: 440.23). **(C, D)** Participant No. 2 under ADP stimulation: (C) Control (MFI: 303.23) and (D) Wild-type (MFI: 421.57). These plots demonstrate a continuous distribution of CD62P fluorescence rather than a bimodal pattern. Agonist stimulation caused a rightward shift of the entire platelet population (increased MFI) across all participants. Due to the lack of a clear negative/positive separation, MFI was adopted as the most reliable metric to capture the population-wide activation state. ADP: adenosine diphosphate; Ctrl: control; W: wild-type; O: Omicron variant; MFI: mean fluorescence intensity; PE: phycoerythrin.

**Figure S3** Representative platelet aggregation curves.


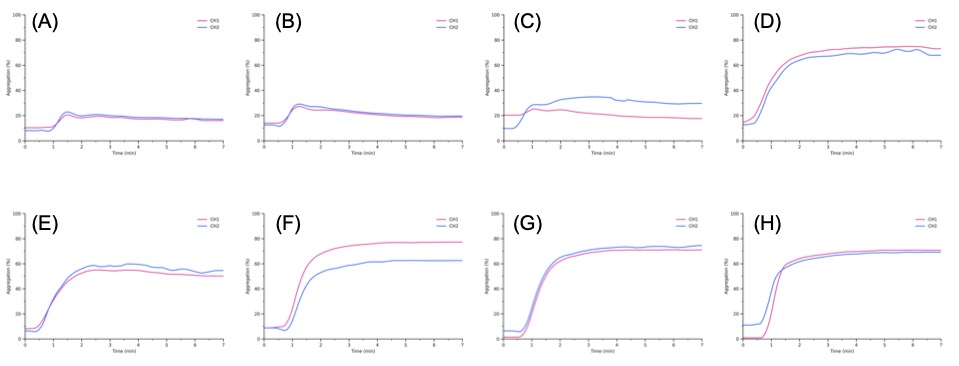


Representative waveforms of platelet aggregation recorded from Participant No. 4. Platelet-rich plasma (PRP) was pre-incubated with BSA (Ctrl), Wild-type spike protein (W), Omicron spike protein (O), or adrenaline (P-Ctrl) for 5 minutes at 37ºC. Subsequently, aggregation was induced by stimulation with **(A-D)** 2 µM of ADP or **(E-H)** 1.4 µM of SFLLRN. Specifically, panels represent: **(A, E)** Ctrl group, **(B, F)** W group, **(C, G)** O group, and **(D, H)** P-Ctrl group. The vertical axis represents aggregation (%), and the horizontal axis represents time (minutes). PRP: platelet-rich plasma; BSA: bovine serum albumin; Ctrl: control (BSA); P-Ctrl: positive control (20 µM adrenaline); ADP: adenosine diphosphate; SFLLRN: protease-activated receptor (PAR)-1 agonist.
