# Supplementary figures and images for: SARS-CoV-2 Omicron variant spike protein maintains the ability to bind and activate human platelets: Comparison with the wild-type
Source: Sci Rep. 2026 Apr 9;16:16753. doi: 10.1038/s41598-026-46081-0 (PMC13223238; doi:10.1038/s41598-026-46081-0)

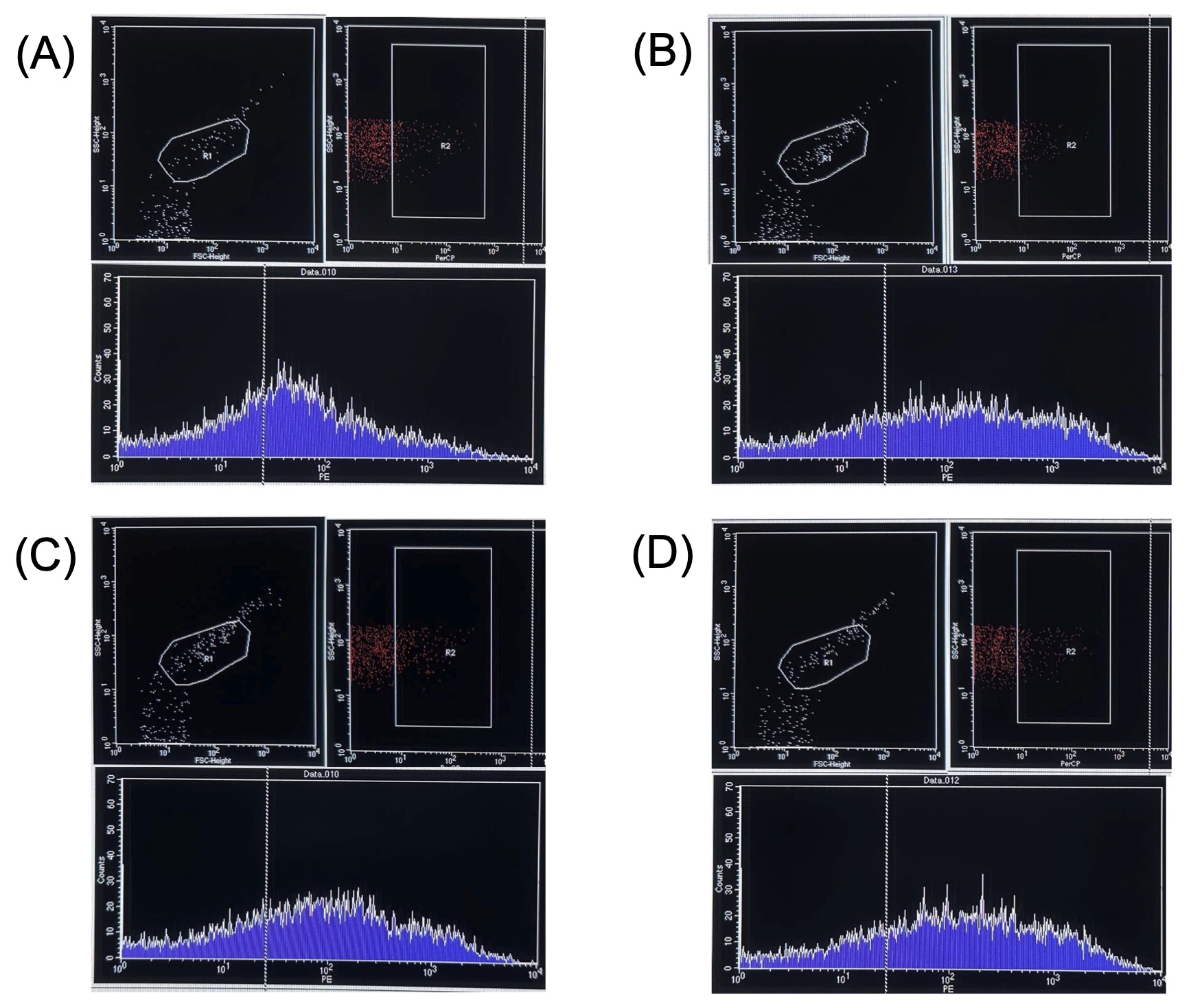

Supplement: Supplementary file 2 — Supplementary Material 1 [file 41598_2026_46081_MOESM2_ESM.jpg]
